# Plasma proteins predict conversion to dementia from prodromal disease

**DOI:** 10.1016/j.jalz.2014.05.1749

**Published:** 2014-11

**Authors:** Abdul Hye, Joanna Riddoch-Contreras, Alison L. Baird, Nicholas J. Ashton, Chantal Bazenet, Rufina Leung, Eric Westman, Andrew Simmons, Richard Dobson, Martina Sattlecker, Michelle Lupton, Katie Lunnon, Aoife Keohane, Malcolm Ward, Ian Pike, Hans Dieter Zucht, Danielle Pepin, Wei Zheng, Alan Tunnicliffe, Jill Richardson, Serge Gauthier, Hilkka Soininen, Iwona Kłoszewska, Patrizia Mecocci, Magda Tsolaki, Bruno Vellas, Simon Lovestone

**Affiliations:** a Institute of Psychiatry, Department of Old Age Psychiatry, King's College London, London, UK; b Department of Neurobiology Care Sciences and Society, Karolinska Institutet, Stockholm, Sweden; c Department of Neuroimaging Genetics, QIMR Berghofer Medical Research Institute, Brisbane, Australia; d University of Exeter Medical School, Exeter University, Exeter, UK; e Proteome Sciences plc, Research & Development, Proteome Sciences plc, Cobham, UK; f Protein Analysis and Detection, EMD Millipore Corporation, St. Charles, MO, USA; g Neurosciences Therapy Area Unit, GlaxoSmithKline Medicines Research Centre, Hertfordshire, UK; h Departments of Neurology & Neurosurgery, Psychiatry, Medicine, McGill Centre for Studies in Aging, Verdun, Canada; i on behalf of GenADA consortium; j Department of Neurology, University of Eastern Finland and Kuopio University Hospital, Kuopio, Finland; k Department of Old Age Psychiatry and Psychotic disorders, Medical University of Lodz, Lodz, Poland; l Institute of Gerontology and Geriatrics, University of Perugia, Perugia, Italy; m 3rd Department of Neurology, Aristotle University, Thessaloniki, Greece; n Department of Internal Medicine and Geriatric Medicine, INSERM U 558, University of Toulouse, Toulouse, France; o Department of Psychiatry, University of Oxford, Oxford, UK; p on behalf of AddNeuroMed consortium

**Keywords:** Plasma, Mild cognitive impairment, Pathology, Alzheimer's disease, Biomarker, Prediction and magnetic resonance imaging

## Abstract

**Background:**

The study aimed to validate previously discovered plasma biomarkers associated with AD, using a design based on imaging measures as surrogate for disease severity and assess their prognostic value in predicting conversion to dementia.

**Methods:**

Three multicenter cohorts of cognitively healthy elderly, mild cognitive impairment (MCI), and AD participants with standardized clinical assessments and structural neuroimaging measures were used. Twenty-six candidate proteins were quantified in 1148 subjects using multiplex (xMAP) assays.

**Results:**

Sixteen proteins correlated with disease severity and cognitive decline. Strongest associations were in the MCI group with a panel of 10 proteins predicting progression to AD (accuracy 87%, sensitivity 85%, and specificity 88%).

**Conclusions:**

We have identified 10 plasma proteins strongly associated with disease severity and disease progression. Such markers may be useful for patient selection for clinical trials and assessment of patients with predisease subjective memory complaints.

## 1 Introduction

Alzheimer's disease (AD) is the most common neurodegenerative disorder of the aging population; usually affecting people over the age of 65 years and resulting in progressive cognitive and functional decline. Detecting AD at the earliest possible stage is vital to enable trials of disease modification agents and considerable efforts are being invested in the identification and replication of biomarkers for this purpose.

Such biomarkers currently include measures of tau and amyloid beta (Aβ) in cerebrospinal fluid (CSF), brain atrophy using magnetic resonance imaging (MRI), and measures of Aβ pathological load using positron emission tomography (PET). All these approaches are promising, although molecular imaging is currently a costly procedure available in relatively few centers and lumbar puncture is moderately invasive. Furthermore, repeated measures are problematical in both cases.

Blood (plasma) on the other hand is a more accessible biofluid suitable for repeated sampling. This led many groups including ours to investigate the potential of a diagnostic signal in blood. Using a case-control study design with a gel-based approach (two-dimensional gel electrophoresis and liquid chromatography tandem mass spectrometry) two proteins (complement factor H [CFH] and alpha-2-macroglobulin [A2M]) were observed as potential markers of AD [1], both of which were subsequently replicated by independent groups [2], [3]. In the same study we observed changes in serum amyloid P (SAP), complement C4 (CC4), and ceruloplasmin, all of which have been implicated in AD pathogenesis [4], [5], [6]. However, case-control studies are problematical when there is a long prodromal disease phase. In such instances a large proportion of apparently normal controls already harbors the disease processes and hence may already have a peripheral biomarker disease signature. To overcome the limitations of case-control design, we searched for proteins associated with surrogates of disease severity (hippocampal atrophy and clinical progression), and identified Clusterin as a marker associated with both these surrogate measures [7]. Building on this “endophenotype” discovery approach we subsequently found transthyretin (TTR) and apolipoprotein A1 (ApoA1) to be associated with faster declining AD subjects and increased plasma apolipoprotein E (ApoE) levels related to increased Aβ burden in the brain [8], [9].

These studies, and those from other groups, have identified a set of proteins that might act as biomarkers relevant to AD. However such findings require replication, in large studies, ideally using samples drawn from more than one cohort source and using a platform that enables multiplexing. We therefore developed multiplex panels using our discovery proteins together with additional putative candidate biomarkers that have been implicated in AD and neurodegeneration (Supplementary Table S1).

The aims of the current study were (1) to validate a set of blood-based biomarkers in a large multicenter cohort with specified *a priori* outcome variables of the disease endophenotype measure of atrophy on MRI and of clinical severity and (2) to determine the accuracy of a multiplexed panel of disease relevant biomarkers in predicting conversion of mild cognitive impairment (MCI) to dementia in a defined time period.

## 2 Methods

### 2.1 Subjects and clinical classification

Plasma samples from AD, MCI subjects and elderly nondemented controls were selected from three independent studies. AddNeuroMed (ANM) a multicenter European study [10], Kings Health Partners-Dementia Case Register (KHP-DCR) a UK clinic and population based study and Genetics AD Association (GenADA) a multisite case-control longitudinal study based in Canada [11]. The diagnosis of probable AD was made according to *Diagnostic and Statistical Manual for Mental Diagnosis*, fourth edition and National Institute of Neurological, Communicative Disorders and Stroke–Alzheimer's disease and Related Disorders Association criteria. MCI was defined according to Petersen criteria [12]. Standardized clinical assessment included the Mini-Mental State Examination (MMSE) for cognition and for global levels of severity the Clinical Dementia Rating (ANM and KHP-DCR only). The human biological samples were sourced ethically and their research use was in accord with the terms of the informed consents.

In total we examined plasma samples from 1148 subjects: 476 with AD, 220 with MCI, and 452 elderly controls with no dementia (Table 1). The APOE single nucleotide polymorphisms (SNPs) rs429358 and rs7412 were genotyped using Taqman SNP genotyping assays (determined by allelic discrimination assays based on fluorogenic 5′ nuclease activity) and the allele inferred.

**Table 1:** Subject demographics

	Control	MCI		AD	Significance
		MCI_nc_	MCI_c_		
N	452	169	51	476	
Age (yrs)	75.6 (±6.3, 53–93)	76.3 (±5.7, 65–90)	76.2 (±6.9, 56–89)	77.0 (±6.4, 58-96)	*P* = .012†
Sex (%, female)	55.6%	50.1%	49.1%	49.4%	*P* = .277
APOE genotype (%, e4+)	28%	35%	55%	59%	*P* < .001†
MMSE	29.0 (±1.2, 22–30)	26.9 (±2.9, 0–30)	26.3 (±2.1, 18-30)	20.8 (±5.4, 0–30)	*P* < .001∗
CDR (sum of boxes)	0.18 (±0.4, 0–3)	1.82 (±0.9, 0–4.5)	2·41 (±0.9, 0.5–5)	4.04 (±3.2, 0–20)	*P* < .001∗

Abbreviations: MCI, mild cognitive impairment; MCI_nc_, mild cognitive impairment non-converter; MCI_c_, mild cognitive impairment converter; AD, Alzheimer's disease; APOE, apolipoprotein E; MMSE, Mini-Mental State Examination; CDR, clinical dementia rating; mean (±standard deviation, range), analysis of variance was performed and if significant a Tukey's post hoc comparison was carried out.

∗ Significance across all three groups.

† Control compared with AD.

### 2.2 Cognitive decline

Cognitive decline, as determined by the slope of change in cognition, was calculated for a subset of AD subjects (n = 342) who had a minimum of three separate MMSE assessments. The rate of cognitive decline was calculated separately for ANM because it had a different following up interval (every 3 months for 1 year) in comparison to DCR and GenADA, which were followed up yearly for a period of at least 3 years. Linear mixed effect models were generated using the package “nlme” in R. We estimated the rate of change using a multilevel linear model with random intercepts and random slopes adjusted for subject and center level clustering. Covariates including age at baseline, gender, APOE ε4 allele presence, and years of education were investigated for their effect on the rate of decline. Age at baseline and years of education had a significant effect on the rate (*P* value < .05) and thus were included as fixed effects in the final model. The slope coefficient obtained from the final model for each sample was then used as a rate of cognitive change, defined as the change in MMSE score per year.

### 2.3 Magnetic resonance imaging

High-resolution sagittal 3D T1-weighted Magnetization prepared rapid gradient-echo (MPRAGE) volume (voxel size 1.1 × 1.1 × 1.2 mm³) and axial proton density/T2-weighted fast spin echo images were acquired on 1.5 T MRI scanners for 476 of the subjects (179 control, 123 MCI, and 174 AD) as previously reported [13]. The MPRAGE volume was acquired using a custom pulse sequence specifically designed for the Alzheimer's Disease Neuroimaging Initiative (ADNI) study to ensure compatibility across scanners [14]. Full brain and skull coverage were required for all MR images according to previously published quality control criteria [13], [15]. Image analysis was carried out using the FreeSurfer image analysis pipeline (version 5.1.0) to produce regional cortical thickness and subcortical volumetric measures as previously described [16], [17]. This segmentation approach has been previously used for analysis in imaging proteomic studies [18] and AD biomarker discovery [16]. All volumetric measures from each subject were normalized by the subject's intracranial volume, whereas cortical thickness measures were used in their raw form [19]. Measures of hippocampal volume, entorhinal cortex volume, and ventricular volume were chosen as MRI endophenotypes of AD. For the evaluation of hippocampal atrophy the MRI data were stratified into high and low atrophy for the MCI group based on their median volumetric measures.

### 2.4 Immunoassay–Luminex measurement

All candidate proteins were measured using multiplex bead assays (Luminex xMAP) (Supplementary S1) incorporated in 7 MILLIPLEX MAP panels (Supplementary S2 and S3) run on the Luminex 200 instrument according to manufacturer's instructions.

### 2.5 Data preprocessing

Before statistical analysis, we examined the performance of each assay using quality checks (QC) as outlined in the Supplementary Material. Median fluorescent intensity (MFI) was measured using xPONENT 3.1 (Luminex Corporation) and exported into Sigma plot (Systat Software; version 12) for estimation of protein concentrations using a five-parameter logistic fit. Briefly, all analytes that passed QC checks based on the following four criteria (standard curve linearity, intra-assay coefficient of variation [CV], interassay coefficient of variation for reference sample, and percentage of missing data; Supplementary Material S4) were taken forward for further analysis.

### 2.6 Statistical analysis

#### 2.6.1 Univariate analysis

Univariate statistical analysis was performed in SPSS 20 (IBM). All raw MFI measures were log_10_ transformed to achieve normal distribution. Covariates including age, gender, plasma storage duration (days), and center were investigated. We found that most proteins were significantly affected by these covariates and therefore values were adjusted using a generalized linear regression model (GLM). All subsequent analysis was performed on the GLM adjusted data. Partial correlation (adjusting for APOE genotype) analysis was performed to examine associations with either structural MRI brain imaging or cognition assessments. Correlations were performed separately within diagnostic groups due to the discrete nature of the clinical scores across all groups. The proteins were also analyzed individually for their association with disease phenotypes: disease status (AD vs. control) via analysis of covariance (adjusting for APOE genotype). Multiple linear regressions were performed to test how combinations of proteins could predict hippocampal volume.

#### 2.6.2 Classification analysis

Class prediction and attribute selection were performed using WEKA (University of Waikato). Naive Bayes Simple algorithm was used with default settings unless stated otherwise. The data set was randomly split into 75% train and 25% test for the MCI-converter (MCI_c_) and MCI-nonconverter (MCI_nc_) groups. Attribute selection was performed using the Classifier Subset Evaluator with the best first search method on the training data. Five iterations of attribute selection were performed ranked by times observed. Proteins seen greater than three or more times were taken forward as predictor variables (Table 3). Any class imbalance was overcome by applying the Synthetic Minority Oversampling Technique in WEKA.

**Table 2:** Proteins identified as significantly associated with structural brain MRI measures in the (A) MCI group and (B) AD group

(A) MCI group				
MRI brain region	Protein	Correlation coefficient∗	Significance (two-tailed)	df
Ventricular volume	Clusterin	0.23	0.01	115
	RANTES	−0.19	0.03	116
Mean hippocampal volume	Clusterin	−0.38	0.00	115
	NSE	0.22	0.02	116
Right Entorhinal thickness	Clusterin	−0.22	0.02	115
Left Entorhinal thickness	Prealbumin	−0.20	0.04	109
Mean Entorhinal volume	n/a	n/a	n/a	n/a
Mean Entorhinal Thickness	n/a	n/a	n/a	n/a
Whole Brain Volume	Clusterin	−0.25	0.01	118
	NSE	0.21	0.02	119
	RANTES	0.19	0.04	119

(B) AD group				
MRI brain region	Protein	Correlation coefficient∗	Significance (2-tailed)	df
Ventricular volume	A1AT	0.24	0.01	119
	NSE	0.16	0.03	169
Mean hippocampal volume	BDNF	−0.21	0.02	123
	ApoC3	−0.18	0.02	168
	ApoA1	−0.15	0.04	169
	ApoE	−0.15	0.05	169
Right entorhinal thickness	n/a	n/a	n/a	n/a
Left entorhinal thickness	n/a	n/a	n/a	n/a
Mean entorhinal volume	ApoC3	−0.20	0.01	168
	ApoE	−0.18	0.02	169
Mean entorhinal thickness	ApoC3	−0.22	0.01	168
	ApoA1	−0.21	0.01	169
	ApoE	−0.2	0.01	169
	Prealbumin	−0.15	0.05	158
Whole-brain volume	ApoE	−0.19	0.02	145
	ApoA1	−0.19	0.02	145
	AB40	0.17	0.04	141

Abbreviations: MRI, magnetic resonance imaging; MCI, mild cognitive impairment; AD, Alzheimer’s disease; NSE, neuron-specific enolase; RANTES, Regulated on Activation, Normal T Cell Expressed and Secreted; n/a, no significant association observed.

∗ Pearson's correlation coefficient.

**Table 3:** Proteins observed in the feature selection

Protein	No. of times observed in feature selection	Protein	No. of times observed in feature selection
**Transthyretin**	5	CathepsinD	1
**Clusterin**	4	ApoE	1
**Cystatin C**	4	SAP	0
**A1AcidG**	4	Ceruloplasmin	0
**ICAM1**	4	NCAM	0
**CC4**	4	NSE	0
**PEDF**	4	VCAM1	0
**A1AT**	4	A2M	0
**APOE genotype**	3	B2M	0
**RANTES**	3	BDNF	0
**ApoC3**	3	CFH	0
PAI-1	2	ApoA1	0
CRP	2	Ab40	0

Abbreviations: NSE, neuron-specific enolase; SAP, serum amyloid P; CFH, complement factor H; CRP, C-reactive protein.

NOTE. Ranked according to the number of times a protein was observed in the feature selection. Proteins highlighted in bold were taken forward as the predictors for MCI conversion.

#### 2.6.3 Cut-off point analysis

Untransformed protein concentrations on the full data set (n = 169, MCI_c_, and n = 51, MCI_nc_) were binarised at different cut-off points using the upper and lower quartile ranges and the percentile rank. A minimum of three cut-off concentrations were tested per protein. Logistic regression analysis was performed on individual cut-off concentrations and selected based on their accuracy of predicting conversion.

## 3 Results

### 3.1 Study participants

The demographic and clinical characteristics of participants from the three cohorts are presented in Table 1. The AD group were marginally, but significantly older than controls (AD: mean 77 years, controls: 75 years, *P* = .01). The frequency of the APOEε4 allele was higher in MCI and AD groups than in controls.

### 3.2 Plasma proteins and brain atrophy

Of the 26 proteins measured only two proteins were found to be significantly different between AD and controls (ApoE: *F* = 6.5, *P* < .001; CFH: *F* = 6.1, *P* < .001). However, using partial correlation, and adjusting for APOE, we identified a number of plasma proteins that were significantly associated with atrophy using MRI measures of one or more of the brain regions; hippocampus, entorhinal cortex, ventricles, and whole-brain volume in the disease groups (Table 2A and B). Controlling for multiple testing, only Clusterin (MCI group: *P* < .001) and ApoE (AD group: *P* = .0014) remained significant.

We then set out to identify proteins that collectively would predict disease progression, as represented by the surrogate of hippocampal atrophy, in a predisease group of MCI. Using multiple linear regression analysis, we identified six proteins (Clusterin, regulated on cctivation, normal T cell expressed and secreted [RANTES], neuron-specific enolase [NSE], TTR, vascular cell adhesion molecule 1 [VCAM-1], and SAP) that predicted 19.5% (*P* = .006) of hippocampal volume in subjects with MCI. We observed a different combination of proteins associated with atrophy in the AD group. Using linear regression analysis, seven proteins (APOA1, alpha-1 antitrypsin [A1AT], ApoC3, brain-derived neurotrophic factor [BDNF], AB40, plasminogen activator inhibitor-1 [PAI-1], and NSE) in the AD group were able to predict 11.9% (*P* = .039) of hippocampal volume.

In summary we found an association of Clusterin with greater atrophy, and a trend toward reduced RANTES, NSE, and TTR levels in the MCI group. In the AD group A1AT, NSE, ApoC3, ApoA1, ApoE, and BDNF plasma levels were increased in subjects with increased atrophy.

### 3.3 Plasma proteins clinical cognition and cognitive decline

We examined the relationship between these proteins and disease severity as measured by cognition at the time of sampling and by the rate of change in cognition. In the MCI group at the point of sampling, both ApoE and C-reactive protein (CRP) negatively correlated with MMSE (ApoE: r = −0.15, *P* = .001; CRP: r = −0.186, *P* = .007).

In the AD group at the point of sampling ApoE, CFH, neural cell adhesion molecule [NCAM], AB40, A1AcidG, and Clusterin were all negatively correlated with MMSE (ApoE: r = −0.150, *P* = .001; CFH: r = −0.104, *P* = .026; NCAM: r = −0.114, *P* = .014; AB40: r = −0.161, *P* = .001; A1AcidG: r = −0.135, *P* = .004; Clusterin: r = −0.135, *P* = .004).

Furthermore, we assessed the association of the proteins with longitudinal prospective MMSE change in the AD group. Three proteins, NCAM, soluble receptor for advanced glycation end products [sRAGE], and intercellular adhesion molecule [ICAM], were significantly associated with the rate of change in cognition; NCAM and sRAGE were both negatively correlated (NCAM: r = −0.129, *P* = .0018; sRAGE: r = −0.125, *P* = .029), whereas ICAM was positively correlated (ICAM: r = 0.108, *P* = .047).

### 3.4 Protein biomarkers to predict disease conversion: MCI to AD

In summary we confirm that a number of proteins, previously identified as putative markers of AD, correlated with disease severity, measured by MRI or severity of cognitive impairment not only in disease but in the predisease state of MCI. We therefore reason that if these proteins are reflecting pathological load, they may also be markers predictive of conversion from predisease states such as MCI to clinical dementia.

**Fig. 1:**
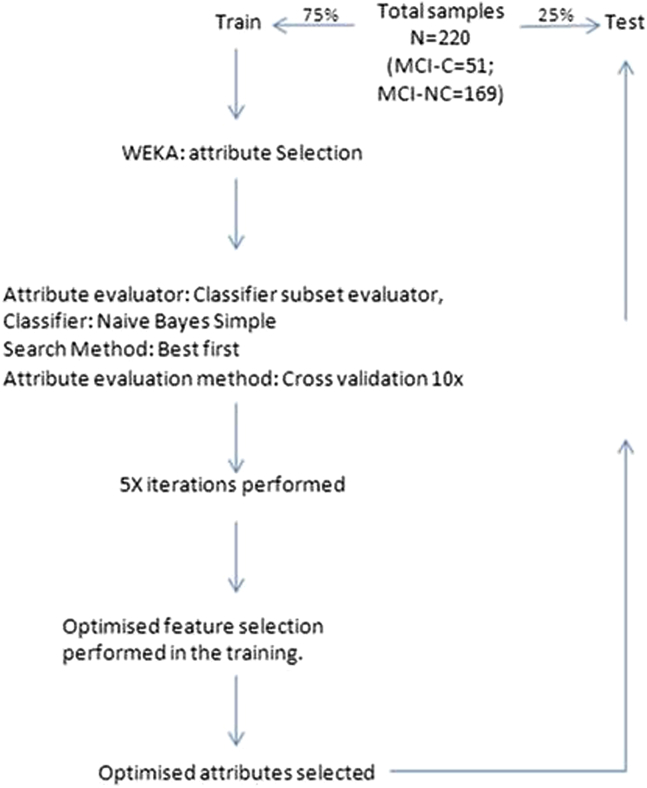
Feature selection workflow used to select the best attributes for mild cognitive impairment converter (MCI_c_) classification.

**Fig. 2:**
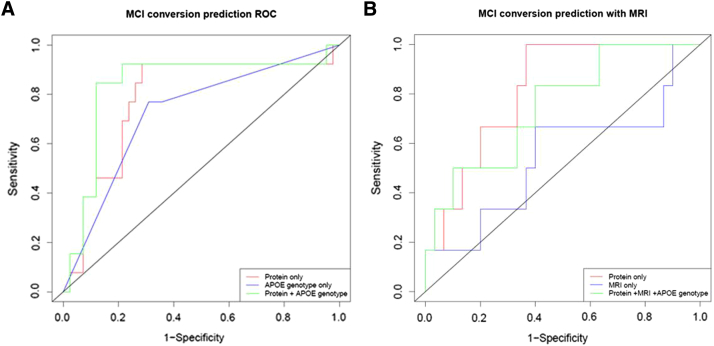
Receiver operating characteristic (ROC) curves obtained for the test set for (A) three models (proteins only, proteins + apolipoprotein E [APOE], and APOE only) from the full mild cognitive impairment, converter and nonconverter (MCI_c_ and MCI_nc_) data sets and (B) for the test set for three models (proteins only, proteins + APOE + magnetic resonance imaging [MRI], and MRI only) in the subset with protein plus MRI imaging data.

To test this, we used a machine learning approach (Naive Bayes Simple) with feature selection on a training data set (Figure 1) and then applied this to a test set. The average time of conversion of MCI to AD was approximately 1 year (375 days, SD = 23 days). Ten proteins (TTR, Clusterin, cystatin C, A1AcidG, ICAM1, CC4, pigment epithelium-derived factor [PEDF], A1AT, RANTES, ApoC3) plus APOE genotype had the greatest predictive power (Table 3). The receiver operating curve characteristic (ROC) for the independent test set is shown in Figure 2A. The ROC area under the curve (Table 4A) of the test set was 0.78 (protein only) and 0.84 (protein + APOE genotype). To test the accuracy, we investigated three different sensitivity cut-off points at 30%, 50%, and 85%. The optimal accuracy was observed at the 85% sensitivity with the test achieving an accuracy of 87% with a specificity of 88%.

**Table 4:** Characteristics of the ROC curve for (A) the full data set without MRI and (B) ROC curve characteristics for the subset with MRI imaging data

(A) ROC characteristics without MRI							
Classification model	Sensitivity cut-off %	SN %	SP %	PPV %	NPV %	ACC %	ROC (AUC)
Protein + APOE	30	30.8	92.9	57.1	81.3	87.2	0.84
Protein only	30	30.8	92.9	57.1	81.3	87.2	0.78
Protein + APOE	50	53.9	88.1	58.3	86.1	80.0	0.84
Protein only	50	43.8	84.6	53.9	78.6	72.7	0.78
Protein + APOE	85	84.6	88.1	68.8	94.9	87.2	0.84
Protein only	85	84.6	71.4	47.8	93.8	74.5	0.78

(B) ROC characteristics with MRI							
Classification model	Sensitivity cut-off %	SN %	SP %	PPV %	NPV %	ACC %	ROC (AUC)
Protein + APOE + MRI	30	33.3	96.7	66.7	87.9	86.1	0.75
Protein only	30	33.3	93.3	50.0	87.5	83.3	0.82
MRI only	30	33.3	80.0	25.0	85.7	72.2	0.54
Protein + APOE + MRI	50	50.0	90.0	50.0	90.0	83.3	0.75
Protein only	50	50.0	86.7	42.9	89.7	80.6	0.82
MRI only	50	50.0	63.3	21.3	86.4	61.1	0.54
Protein + APOE + MRI	85	83.3	60.0	29.4	94.7	63.9	0.75
Protein only	85	83.3	66.7	33.3	95.2	69.4	0.82
MRI only	85	83.3	13.3	16.1	80.0	25.0	0.54

Abbreviations: ROC, receiver operating characteristic; MRI, magnetic resonance imaging; SN, sensitivity; SP, specificity; PPV, positive predictive value; NPV, negative predictive value; ACC, accuracy; AUC, ROC area under curve for the protein and APOE classifiers.

NOTE. Three different sensitivity cut-off points were investigated.

We then investigated whether combining structural MRI data with these 10 proteins observed in the MCI conversion data would improve classification accuracy. MRI brain measures for a subset of subjects were combined with the protein data and the Naive Bayes algorithm was applied. In this smaller data set the proteins alone performed very well when tested at the three different sensitivity cut-offs (cut-off: accuracy; 30%: 83.3%, 50%: 80.6%, 85%: 69.4%). The addition of MRI data only marginally improved the accuracy at two cut-off points (cut-off: accuracy 30%: 86%; 50%: 83%) but reduced it at the 85% sensitivity cut-off to 64% (Figure 2B and Table 4B).

### 3.5 Concentration cut-offs points for proteins predicting MCI to AD

Individual protein cut-off values were derived for the 10 proteins identified by feature selection in the MCI conversion model. Values predictive of conversion to AD were; ApoC3< 105.5 μg/ml, TTR < 222 μg/ml, A1AT< 9.5 μg/ml, PEDF > 10.7 μg/ml, CC4 > 78.5 μg/ml, ICAM-1< 99.72 ng/ml, RANTES< 33.8 ng/ml, A1AcidG > 768.3 μg/ml, Cystatin C < 3.21 μg/ml, Clusterin > 402 μg/ml. Logistic regression was applied to test the 10 protein cut-off concentrations and APOE genotype, the overall model accuracy was 94.9%, with a sensitivity 73.6% and specificity of 94.9% when using the full data set.

## 4 Discussion

Previous studies by our group using data-driven pan-proteomic approaches have identified a number of proteins as diagnostic [1] progression [7], [20] and markers of disease severity [18]. The advent of high throughput multiplex platforms facilitates the replication of such findings and raises the potential of high throughput multiplexed markers for use in clinical practice and in clinical trials [21], [22], [23]. In this study we have used a multiplex antibody capture platform to determine if our putative biomarkers are associated with early disease stages and might have value as prognostic markers. Using MRI as a surrogate of disease pathology we identified a number of proteins that were associated with atrophy either early in the disease process (MCI) or in established dementia.

This approach of using MRI as a proxy for *in vivo* pathology has previously been shown to be useful in biomarker discovery, including in our study identifying Clusterin as a putative marker of disease [7]. In this study we identified RANTES, NSE, and transthyretin, in addition to Clusterin, to be associated with cortical atrophy in the MCI group, with Clusterin showing the strongest correlation with all brain regions assessed. The other proteins have previously been implicated in AD. RANTES, also known as chemokine ligand 5, is a protein known to have an active role in recruiting leukocytes into inflammatory sites. We find a negative association between RANTES and ventricular volume, suggesting a decreased level with increased disease related pathology; the opposite to previous reports in neurodegeneration [24], [25], [26]. One possible explanation might be that because we observe RANTES association with atrophy only in MCI and not in AD, perhaps a decrease early in disease process is followed by an increase. Similar findings have been previously reported for other proteins [27] and we also observe a similar relationship with NSE, the second protein we observe in association with brain atrophy. This protein is thought to be an indicator of acute neuronal damage [28], [29] and has been associated with AD in some but not all previous studies [30], [31]. We find a positive association between NSE and volume of hippocampus and whole brain in MCI subjects, but in the AD group we find a positive association instead with ventricular volume. This inverse relationship with atrophy in predisease and then positive correlation with atrophy in disease suggests to us, that like RANTES, NSE might be decreased in early disease stages (i.e. MCI) with a rebound elevation in established AD.

In established AD we observe a different set of proteins associated with disease severity as measured by atrophy on MRI, in line with this concept of disease phase specific biomarkers. A number of these belong to the group of apolipoproteins (ApoE, ApoC3, and ApoA1). We found these proteins were negatively associated with hippocampal, entorhinal cortical, and whole-brain volumes. The roles of apolipoproteins in neurodegenerative disorders have been studied extensively because the discovery that APOE was a major susceptibility gene for AD [32], [33]. In the peripheral system, ApoE serves in the transport of triglycerides, phospholipids, and cholesterol into cells [34]. The literature on ApoE is conflicting with some groups reporting lower ApoE in AD [35], [36], with others showing increased levels [37], [38]. ApoE plasma measurements derived from this study have been recently published and are in agreement with the findings from the North American Alzheimer's Disease Neuroimaging Initiative (ADNI), which reports an APOE genotype effect [39].

Our present findings suggest that we have identified a panel of plasma biomarkers, associated with neuroimaging measures of disease, which may serve as readily accessible markers of early disease severity. Moreover, we identify a set of 10 protein biomarkers that can prospectively predict disease conversion from MCI to AD within a year of blood sampling. These results are supported by other evidence that plasma proteins can have a role in early disease detection, with inflammatory proteins in particular being identified as possible predictors of conversion from MCI [23], [40]. It is important to note that when attempting to compare such biomarker studies, the lack of standardized reagents, particularly antibodies may result in different outcomes reflecting technical differences between analytical platforms more than disease biology. Therefore our ability to replicate these proteins using an orthogonal approach (mass spectrometry in discovery, multiplexed immune capture in replication) makes these findings particularly powerful and robust. Moreover, combining MRI with protein measures did not improve predictive power in contrast to previous studies where CSF marker performance was improved in combination with MRI [41].

Although this study is built on findings from previous discovery-led and replicated findings, further replication will be needed. Ideally such replication should be in large, longitudinal, population-based cohorts. Such a study would be able to address potential confounds of the data reported in this study including site-specific effects and representativeness of the cohorts. Further studies will also be needed to address specificity. The markers we have identified are often altered in other disease areas–inflammation, cardiovascular, respiratory, dental, and others–and it will be important to distinguish the relative overlap and confounding by these diseases. However, although the protein participants in the panel we have identified are often altered in other disease states, these diseases are all different and therefore the panel itself may show specificity even if the participants do not. This remains to be determined. It also remains to be seen whether the panel we have identified is specific to AD or shows biomarker utility in relation to other dementia syndromes. Although we used an assessment protocol that we have previously shown is highly accurate in distinguishing AD from other dementias based on post-mortem confirmation, it will be interesting in due course to correlate the behavior of our panel to specific markers of dementia pathology such as biochemical or imaging measures of Aβ and tau.

In summary, using a multiplexing approach we have validated a plasma protein panel as a marker reflecting disease severity and for predicting disease progression within three large multicenter cohorts. Such a marker set may have considerable value in triaging patients with early memory disorders, to other more invasive approaches such as molecular markers in CSF and PET imaging, in clinical trials and possibly in clinical practice.

**Research in context**

1. Systematic review: We searched PubMed up to February 2014 using the keywords, Alzheimer's disease (AD), plasma, prediction, pathology, mild cognitive impairment, and MCI to AD conversion.
2. Interpretation: This is the largest (n = 1148) multicenter plasma validation study based on previous discovery candidate biomarkers. In addition, we identified markers that are strongly associated with disease endophenotype measures based on magnetic resonance imaging and clinical severity. Moreover, these biomarkers can prospectively predict disease conversion from MCI to AD with an accuracy of 87% exceeding that of any previous reported plasma biomarkers. Our findings suggest the potential role of these biomarkers detected in plasma as indirect indicators of AD pathology, and their utility as predictors for future disease conversion.
3. Future directions: To validate the clinical utility of the current study results, an independent study is required of an equal or greater size to test the accuracy of this panel of biomarkers.

## Figures and Tables

**Table S1:** Overview of proteins investigated in the current study

Protein name	Method	Study design	Reported findings	References
A2M	2-DGE; LC-MS/MS	AD vs. control	↑ AD	Hye et al. 2006
SAP	2-DGE; LC-MS/MS	AD vs. control	↑ AD	Hye et al. 2006
CFH	2-DGE; LC-MS/MS	AD vs. control	↑ AD	Hye et al. 2006, Cutler et al. 2008
Complement C4	2-DGE; LC-MS/MS	AD vs. control	↓ AD	Hye et al. 2006
ApoE	2-DGE; LC-MS/MS and ELISA	PiB PET association	↑ Aβ brain region	Thambisetty et al. 2010
Clusterin	2-DGE; LC-MS/MS and ELISA	Low vs. high brain atrophy	↑ High atrophy	Thambisetty et al. 2011
ApoA1	2-DGE; LC-MS/MS	SCD vs. FCD	↑ FCD	Thambisetty et al. 2011
TTR	2-DGE; LC-MS/MS and ELISA	SCD vs. FCD	↓ FCD	Velayudhan et al. 2012
Ceruloplasmin	2-DGE; LC-MS/MS	AD vs. control	↓ AD	Hye et al. 2006
Aβ 40 (Aβ40)	ELISA	AD vs. control	↑ AD	Mehta et al. 2001, Mayeux et al, 2003
Aβ 42	ELISA	AD vs. control	↓ AD	Hampel et al, 2010, Blennow et al, 2001
A-1-acid glycoprotein	ELISA	AD vs. control	↓ AD	Roher et al, 2010
Alpha1 antitrypsin (A1AT)	ELISA	AD vs. control	↑ AD	Nielsen et al, 2007, Sun et al, 2003
Apo C3	Luminex	E4 carrier vs. noncarrier	↓ AD	Song et al, 2012
BDNF	ELISA	MRI association	↑ Age related white atrophy	Driscoll et al, 2011
	ELISA	AD vs. control	↓ AD	Aisa et al, 2010
	ELISA	AD vs. control	↓ AD	Laske et, 2006
Beta-2-microglobulin	Luminex		↑ AD	Wilson et al, 2012
Cathepsin D	Western blot	AD vs. control	↓ AD	Urbanelli et al, 2008
CRP	Nephelometric detection	SCD vs. FCD	↑ FCD	Locascio et al, 2008
Cystatin C	Immunoturbidimetric assay	AD vs. control	↓ AD	Zhong et al, 2013;
	ELISA	AD vs. control	No change	Sundelöf et al, 2010
ICAM-1	IHC	AD vs. control	↑ AD	Frohman et al, 1991
NCAM	ELISA	AD vs. control	↓ AD	Aisa et al, 2010
NSE	Electrochemiluminescence assay	AD vs. control	No change	Chaves et al, 2010;
	Immunoradiometric assay	AD vs. control	↑ AD	Blennow et al, 1994
PAI-1	ELISA	AD vs. control	↑ AD	Sutton et al, 1994; Akenami et al, 1997
PEDF	2-DGE; LC-MS/MS	AD vs. control	↑ AD	Castano et al, 2006
RANTES	Q-RT-PCR	AD vs. control	↑ AD	Kester et al, 2011
			↑ AD	Tripathy et al, 2011; Reynolds et al, 2007
VCAM-1	ELISA	AD vs. control	↑ AD	Zuliani et al, 2008
sRAGE	ELISA	AD vs. control	↓AD	Emanuele et al. 2005
	ELISA	AD vs. MCI	↓MCI	Chidoni et al. 2008

Abbreviations: A2M, alpha-2-macroglobulin; 2-DGE, two-dimensional gel electrophoresis; LC-MS/MS, liquid chromatography tandem mass spectrometry; SAP, serum amyloid P; CFH, complement factor H; ELISA, enzyme-linked immunosorbent assay; TTR, transthyretin; Aβ, amyloid beta; CRP, C-reactive protein; IHC, immunohistochemistry; NSE, neuron-specific enolase; Q-RT-PCR, quantitative reverse transcription polymerase chain reaction; MCI, mild cognitive impairment; PiB, Pittsburgh compound B; SCD, Slow cognitive decline; FCD, Fast cognitive decline.

## S2: Milliplex MAP assays

Seven Milliplex^®^ MAP multiplex panels (96-well plate format; EMD Millipore) were utilized: human neurodegenerative panel 1 (7-plex) cat.# HNDG1MAG-36K, panel 2 (6-plex) cat.# HNDG2MAG-36K, panel 3 (10-plex) HNDG3-36K, panel 4 (5-plex) HNDG4-36K, human kidney toxicity panel 2 (3-plex) cat.# HKTX2MAG-38K, human neurological disorders magnetic panel 1 (7-plex) cat.# HND1MAG-39K, and panel 2 (4-plex) cat.# HND2MAG-39K.

## S3: Immunoassay protocol

The Luminex xMAP technology (Austin, TX) uses a solid phase approach to analyze multiple proteins. In brief, the xMAP technology is a flow cytometric-based platform that uses microspheres inserted with a ratio of two different fluorescent dyes. In theory, up to 100 differently colored beads can be generated with a theoretical multiplex capacity of up to 100 assays per well of a 96-well plate. The capture antibody is covalently coupled to the bead, and immunoassays are run under standard sandwich immunoassay formats.

The plasma samples were first diluted as recommended in the protocol for each Milliplex MAP assay. Each assay well was first rinsed with 100 μL wash buffer (1× L-WB) before sample loading. Twenty-five microliters of assay buffer was added to either 25 μL controls or samples followed by 25 μL of beads to bring the total volume in each well to 75 μL. The assay plates were incubated for 2 h at room temperature or overnight with agitation on an orbital shaker. The beads in the plate were washed three times with 100 μL wash buffer and incubated for 1 h with 25 μL of biotinylated detection antibody. Twenty-five microliters of fluorescently labeled reporter (streptavidin-PE) molecule was added to the detection antibody for a further 30 min. Finally the assay plate was washed three times with 100 μL of wash buffer and the beads suspended in 100 μL of sheath fluid. All plasma samples were assayed in duplicate and a pooled plasma (Mastermix) sample was included as a positive control in addition to high and low QC per plate.

## S4: Data quality checks (QC) and preprocessing

Fluorescence in each well was measured using Luminex 200 (Austin, TX) instrument and the results were analyzed with the Xponent 3.1 (Luminex) software. Median fluorescent intensity (MFI) values were exported and the characteristics of the individual samples were confirmed by calculating the mean, standard deviation, and coefficient of variation (CV) % from the MFI readouts. All processed data points were then imported into Sigma Plot (Systat, ver. 12). Using a five-parameter logistic curve fitting method the concentrations of the unknown plasma samples and the master mix were calculated.

Any individual samples that recorded a CV>15% for either duplicate was eliminated; if both duplicates were out of range then both data points were excluded.

Individual analytes were then assessed for quality by applying rank according to their performance in the assay (31 plates; 1153 plasma samples) and defined using a scoring system based on four criteria as follows:

Criteria 1. Standard curve rank: 1 = Good quality-within linear section on standard curve and within QC range. 2 = Moderate quality, spread across the linear section on standard curve, clustered either higher or lower than QC. 3 = Poor quality, not on linear section at all, below low QC or higher than top QC.

Criteria 2. Intra-assay CV (%) for QC1 and QC2, CV<30% accepted (point for each QC value).

Criteria 3. Interassay CV (%) for our in-house pooled sample (master mix), CV<30% accepted.

Criteria 4. Missing data defined as samples that could not be reliably intrapolated from the standard curve. (1) MFI values outside the quantifiable range. (2) Technical failure resulting in no MFI value being generated.
